# Leukocytoclastic vasculitis complicating cisplatin + radiation treatment for laryngeal cancer: a case report

**DOI:** 10.1186/s12885-017-3848-6

**Published:** 2017-12-06

**Authors:** Júlia Coelho França Quintanilha, Marília Berlofa Visacri, Laís Sampaio Amaral, Carmen Silvia Passos Lima, Maria Letícia Cintra, Patricia Moriel

**Affiliations:** 1 School of Medical Sciences (FCM), University of Campinas (UNICAMP), 126 Tessália Vieira de Camargo Street, Campinas, SP 13083-8871 Brazil; 2 Faculty of Pharmaceutical Sciences (FCF), University of Campinas (UNICAMP), 200 Cândido Portinari Street, Campinas, SP 13083-871 Brazil

**Keywords:** Leukocytoclastic vasculitis, Cisplatin, Drug-related side effects, Chemotherapy, Oncology, Case report

## Abstract

**Background:**

Leukocytoclastic vasculitis is typically mediated by deposition of immune complexes and is related to many causes, including medication. To the best of our knowledge, leukocytoclastic vasculitis related to cisplatin has not yet been described in the scientific literature.

**Case presentation:**

We report a rare case of leukocytoclastic vasculitis after the first cycle of high-dose cisplatin chemotherapy in a patient with larynx carcinoma. A 48-year-old Caucasian man with larynx carcinoma received a high-dose of cisplatin monochemotherapy (100 mg/m^2^ every 21 days), along with 70 Gy of radiotherapy divided into 35 sessions, as a therapeutic schedule. Twelve days after the first chemotherapy administration and after 8 sessions of radiotherapy (total of 16 Gy), the patient presented with acute onset of palpable purpura in the lower limbs. The patient was hospitalized for 10 days, and during this period, he underwent several examinations to rule out infectious, autoimmune, and neoplastic disorders. A skin biopsy showed leukocytoclastic vasculitis with a positive pattern for IgM and C3, as detected through direct immunofluorescence. Twenty-five days after cisplatin administration, the chemotherapy regimen was changed to carboplatin AUC 5, and the episodes of purpura ceased, reinforcing the hypothesis of an adverse reaction to cisplatin.

**Conclusions:**

Cisplatin can induce leukocytoclastic vasculitis and clinicians should be aware of this potential effect for better case management and diagnosis.

## Background

Leukocytoclastic vasculitis is a small vessel inflammatory disorder typically mediated by deposition of immune complexes [1]. It is primarily characterized by the presence of palpable purpura due to the infiltration and hemorrhage, and the most affected area is the lower region of the legs [2].

There are many causes of leukocytoclastic vasculitis, including infections, malignancies, foreign protein, chemicals, drugs, and associated diseases such as autoimmune disease [3]. Medications cause 10–15% of leukocytoclastic vasculitis cases [2]. Drug-induced vasculitis may be related to (a) antibodies directed against drug-related haptens, (b) direct drug toxicity on vessel walls, (c) autoantibody reacting with endothelial cells, or (d) a cytokine-mediated reaction to the vascular endothelium associated with interferon-gamma and interleukin-6 [4, 5].

Cisplatin is an anti-cancer drug used for the treatment of solid tumors, such as head and neck cancer. To the best of our knowledge, leukocytoclastic vasculitis related to cisplatin has not yet been described in the scientific literature. Here, we present the case of a patient with larynx carcinoma who experienced leukocytoclastic vasculitis after the first cycle of high-dose cisplatin chemotherapy.

## Case presentation

The patient was a 48-year-old Caucasian man, with a history of smoking, alcoholism, tracheostomy, diagnosed with laryngeal moderately differentiated squamous cell carcinoma, and had not previously received tumor treatment. In November 2016, the patient received high-dose cisplatin monochemotherapy (100 mg/m^2^ every 21 days) along with 70 Gy of radiotherapy into 35 sessions in the head and neck region, as a therapeutic schedule. The only comorbidity was gastroesophageal reflux, and home therapy consisted of omeprazole 20 mg and dexamethasone 4 mg daily, and acetaminophen 500 mg if necessary for pain. On the day of cisplatin administration (total dose of 178 mg), the patient received vigorous hydration (3 L of saline solution; 0.9%), diuretics (125 mL of mannitol; 20%), electrolytes (20 mL of potassium chloride, 19.1%; and 10 mL of magnesium sulphate), and prophylaxis for acute emesis (20 mg of dexamethasone plus 24 mg of ondansetron). From days 2–6 following the first infusion the patient received 10 mg metoclopramide and 100 mg dimenhydrinate every 6 h, and 8 mg ondansetron every 12 h orally to prevent nausea and vomiting. Nevertheless, the patient had nausea during the five days following chemotherapy, decreasing his food intake. He did not have diarrhea, vomiting, or fever. Furthermore, the patient did not report any previous episodes of allergy or anaphylaxis.

**Fig. 1:**
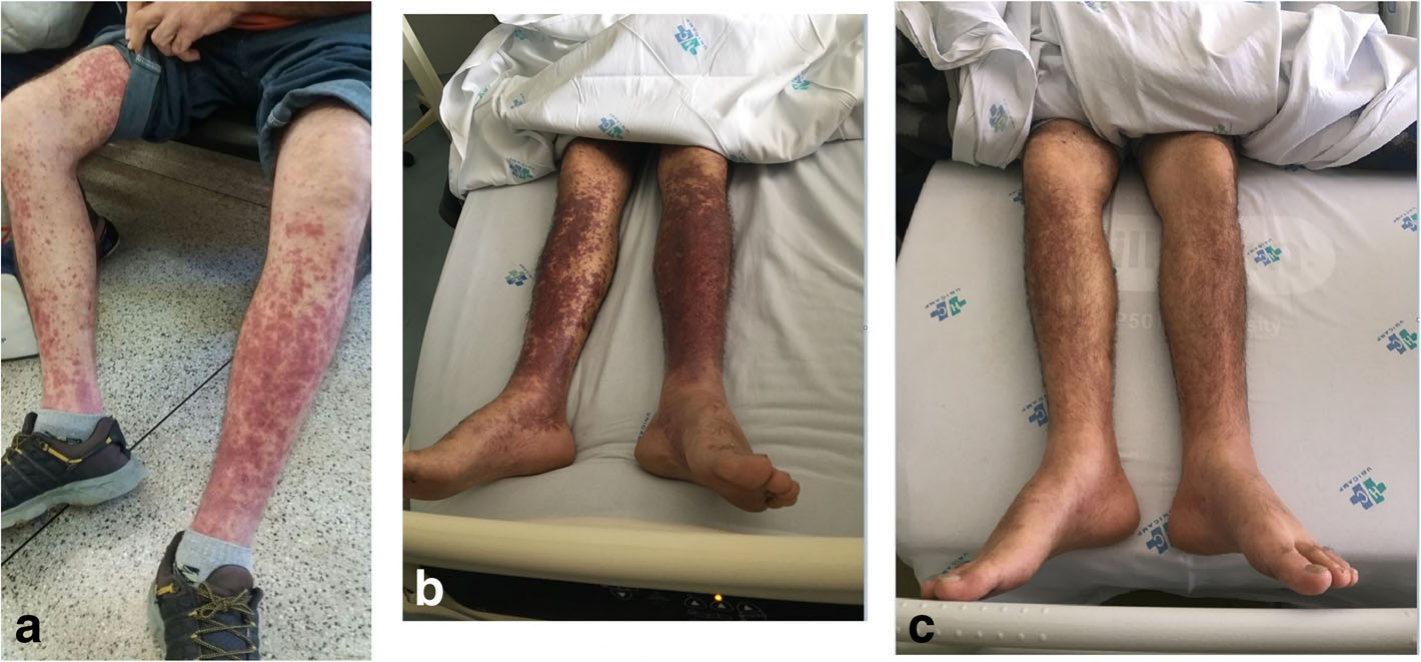
Purpura in the lower limbs. **a** Thirteen days after cisplatin infusion (1 day after first purpura lesion). **b** Fifteen days after cisplatin infusion (3 days after first purpura lesion). **c** Twenty-one days after cisplatin infusion (9 days after first purpura lesion)

**Fig. 2:**
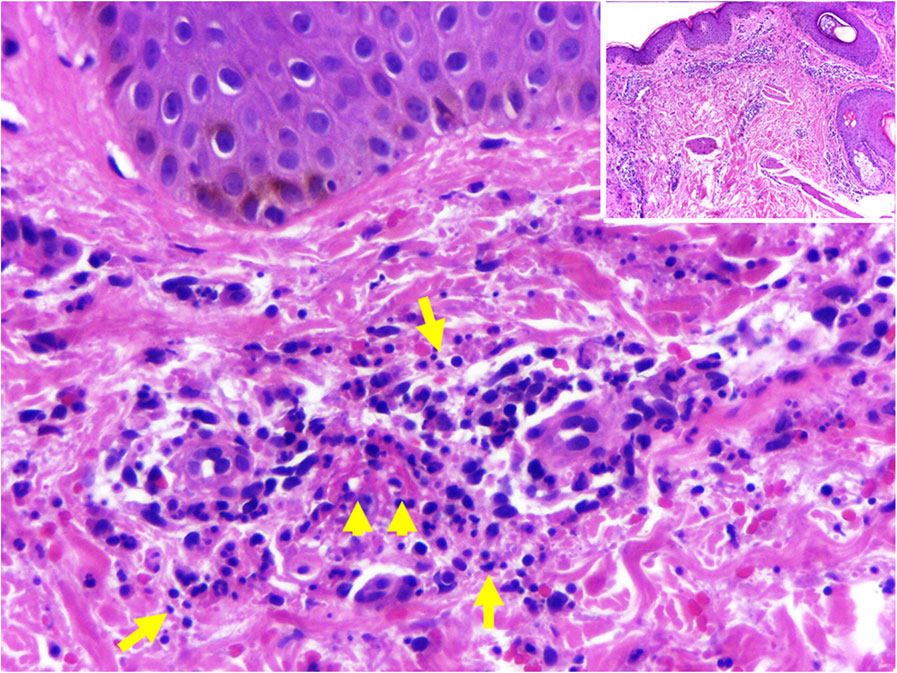
Skin biopsy. Histologically, the walls of vessels (arrowheads) of the superficial dermis were covered by neutrophilic exudate, with marked apoptosis (arrows) of inflammatory cells (leukocytoclasia) and extravasation of red blood cells [HE, original increase ×125 (inset) and × 500]

At 12 days following the first cisplatin infusion and after 8 sessions of radiotherapy (total of 16 Gy), the patient presented with acute onset of palpable purpura in the lower limbs (Fig. 1). On the 13th day following chemotherapy, the patient was hospitalized with suspected meningococcemia and began an antibiotic therapy with intravenous 1000 mg vancomycin every 12 h and 2000 mg cefepime every 8 h. On the 14th day after chemotherapy, the patient underwent a cranial computed tomography scan and cerebrospinal fluid (CSF) analysis that refuted the meningococcemia hypothesis, thus the antibiotic therapy was discontinued. The CSF analysis included adenosine deaminase activity, bacterioscopy, biochemistry, cytology, culture, gram bacterioscopy, IgG dosage, and screening for antitoxoplasma antibodies, mycobacteria, antineoplastic cells, fungi, and anti-cardiolipin antibody. All of the tests had normal or negative results. At the 14th day following the first dose of chemotherapy, a skin biopsy was performed, which revealed neutrophilic exudates coating the walls of the vessels of the superficial dermis, with marked apoptosis of inflammatory cells (leukocytoclasia). There was spilling of red blood cells and degenerative changes in the dermal collagen. The diagnosis of leukocytoclastic vasculitis was therefore performed (Fig. 2). The direct immunofluorescence test revealed mild and granular deposits of IgM and C3 on the wall of some upper dermis vessels. Moreover, blood tests were negative for cryoglobulin, anti-neutrophil cytoplasmic antibody (ANCA), antinuclear antibodies (ANA), hepatitis B and C, and for human immunodeficiency virus.

**Fig. 3:**
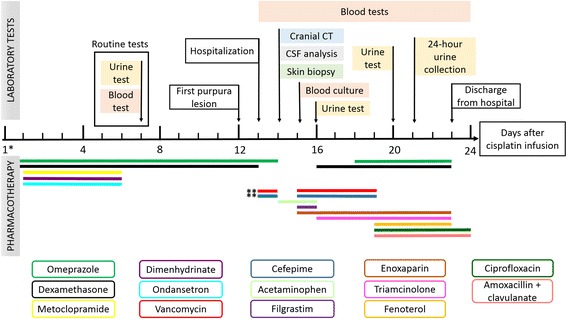
Representative scheme of pharmacotherapy and laboratory tests. CT: computed tomography; CSF: cerebrospinal fluid; *On the day of cisplatin administration (total dose of 178 mg), the patient received vigorous hydration (3 L of saline solution 0.9%), diuretics (125 mL of mannitol 20%), electrolytes (20 mL of potassium chloride 19.1% and 10 mL of magnesium sulphate) and prophylaxis of acute emesis (20 mg of dexamethasone plus 24 mg of ondansetron); **Vancomycin 13th day dose: 1000 mg, after the 15th day: 500 mg; Cefepime 13th day dose: 2000 mg, after the 15th day: 1000 mg

At the 15th day following chemotherapy, a blood test showed marked leukopenia (Table 1); therefore, the patient received 300 mg subcutaneous filgrastim, blood was taken for culture and he restarted prophylactic antibiotic therapy at reduced doses (500 mg vancomycin and 1000 mg cefepime). He also began a regimen of 40 mg daily subcutaneous enoxaparin. At the 16th day following chemotherapy initiation, the patient underwent a urine test, and therapy with 1 mg/g topical triamcinolone daily was initiated owing to radiodermatitis, and therapy with 4 mg oral dexamethasone daily was restarted. At the 18th day, a therapy with 20 mg oral omeprazole daily was also restarted. At the 19th day, the patient received 5 mg/mL (20 mL) fenoterol inhalation therapy every 6 h, and the antibiotic regimen was altered to 500 mg ciprofloxacin every 12 h and 500 mg amoxicillin + 125 mg clavulanate orally every 8 h. Another urine test was performed on the 20th day, and a 24-h urine collection was performed on the 21th day owing to worsening of renal function, as evidenced by increased serum creatinine and blood urea nitrogen (BUN) (Table 2). Discharge from the hospital occurred at the 23th day, once the purpura lesion had recovered and the patient’s renal function started to improve. Figure 3 shows the sequence of pharmacotherapy and laboratory tests.

**Table 1:** Hematological tests performed before and after cisplatin administration

	Basal	D7	D13	D14	D15	D16	D17	D18	D19	D20
Hemoglobin (g/L)	13.9	13.1	11.0	8.9	8.7	8.4	8.9	9.1	9.0	8.3
Leukocytes (×10^3^/mm^3^)	12.82	11.42	4.83	2.50	1.97	2.29	6.18	10.35	10.89	8.64
Neutrophils (×10^3^/mm^3^)	8.20	8.93	3.32	–	–	1.16	–	7.04	7.07	–
Lymphocytes (×10^3^/mm^3^)	3.21	1.54	1.18	–	–	0.73	–	1.14	0.68	–
Monocytes (×10^3^/mm^3^)	1.03	0.87	0.24	–	–	0.33	–	0.72	0.78	–
Eosinophils (×10^3^/mm^3^)	0.00	0.04	0.05	–	–	0.06	–	–	–	–
Basophils (×10^3^/mm^3^)	0.00	0.04	0.04	–	–	0.01	–	–	–	–
Platelets (×10^3^/mm^3^)	312	279	141	114	121	134	156	234	254	251

*Abbreviations:* *D7 – D20* Days after cisplatin administration. *Reference values:* Hemoglobin: 14.0 – 18.0 g/L; Leukocytes: 4.0 – 10.0 × 10^3^/mm^3^; Neutrophils: 2.0 – 8.0 × 10^3^/mm^3^; Lymphocytes: 1.0 – 4.0 × 10^3^/mm^3^; Monocytes: 0.2 – 0.8 × 10^3^/mm^3^; Eosinophils: 0.00 – 0.45 × 10^3^/mm^3^; Basophils: 0.00 – 0.20 × 10^3^/mm^3^; Platelets: 150 – 400 × 10^3^/mm^3^

**Table 2:** Biochemical and other blood tests results performed before and after cisplatin administration

	Basal	D7	D13	D14	D15	D16	D17	D18	D19	D20	D21	D22
INR	–	–	1.00	1.17	–	1.13	1.14	–	–	–	–	–
ESR (mm)	–	–	–	43	–	–	–	–	–	–	–	–
CRP (mg/L)	–	–	–	66	–	–	–	–	–	–	–	–
Glucose (mg/dL)	–	–	–	81	–	–	–	–	–	–	–	–
Creatinine (mg/dL)	0.79	0.97	–	0.87	–	1.49	1.72	1.96	1.93	1.99	1.73	1.55
Ca (mg/dL)	9.3	9.4	–	7.2	–	–	–	–	8.1	–	7.9	–
Mg (mEq/L)	1.65	1.74	–	1.47	–	–	–	–	1.71	–	–	–
Pi (mg/dL)	4.3	4.5	–	3.4	–	4.2	–	–	4.9	–	–	–
K (mEq/L)	4.3	4.7	–	4.2	–	4.6	5.1	5.3	5.6	5.7	5.1	–
Na (mEq/L)	138	136	–	134	–	137	135	132	134	135	135	–
BUN (mg/dL)	31	50	–	35	–	54	70	96	112	108	89	76
Uric Acid (mg/dL)	6.9	6.7	–	7.2	–	–	–	–	8.2	–	–	–
Albumin (g/dL)	3.8	3.8	–	–	–	–	–	–	2.7	–	2.9	–
GGT* (U/L)	50	116	–	50	–	–	–	–	44	–	–	–
TP (g/dL)	6.3	6.6	–	–	–	–	–	–	5.5	–	–	–
C3 (g/L)	–	–	–	0.49	–	–	–	–	–	–	–	–
C4 (g/L)	–	–	–	0.06	–	–	–	–	–	–	–	–
RF (UI/mL)	–	–	–	–	< 10.6	–	–	–	–	–	–	–

*Abbreviations:* *D7 – D22* Days after cisplatin administration, *INR* International normalized ratio, *ESR* Erythrocyte sedimentation rate, *CRP* C-reative protein, *BUN* Blood urea nitrogen, *GGT* Gammaglutamyltransferase, *TP* Total proteins, *RF* Rheumatoid factor; *ALP (alkaline phosphatase), *AST* (aspartate aminotransferase), *ALT* (alanine aminotransferase) and total bilirubin remained within 1.5 of the upper limits of normal. *Reference values:* INR: <1.25; ESR: <10 mm; CRP: ≤3 mg/L; Glucose: 74 – 99 mg/dL; Creatinine: 0.84 – 1.25 mg/dL; Ca: 8.8 – 10.6 mg/dL; Mg: 1.5 – 2.1 mEq/L; Pi: 2.5 – 4.5 mg/dL; K: 3.5 – 5.1 mEq/L; Na: 135 – 145 mEq/L; BUN: 17 – 43 mg/dL; Uric acid: 3.5 – 7.2 mg/dL; Albumin: 3.5 – 5.2 g/dL; GGT: 9 – 64 U/L; TP: 6.6 – 8.3 g/dL; C3: 0.90 – 1.80 g/dL; C4: 0.10 – 0.40 g/dL; RF: <15.00 UI/mL

All the results of blood tests done prior to (basal) and following cisplatin administration are described in Table 1 (hematological tests) and Table 2 (biochemical and other blood tests). We observed that the majority of hematological parameters (hemoglobin, leukocytes, neutrophils, lymphocytes, monocytes, and platelets) decreased after cisplatin chemotherapy and during the hospitalization, and began to increase on day 16 and 17, due to filgrastim administration. Eosinophils and basophils increased after cisplatin infusion; this was observed even on the blood test performed prior to the appearance of purpura lesion (on day 7). Regarding the renal parameters, creatinine and BUN increased primarily on day 16 (3 days after hospitalization) probably due to vancomycin administration, since these parameters began to decrease after the change to the antibiotic regimen used. The hepatic parameters were slightly altered after hospitalization only in relation to albumin and total proteins. The international normalized ratio parameter, a global standard for prothrombin time, was within the reference values. The erythrocyte sedimentation rate (ESR) and c-reactive protein (CRP) levels were altered in the test performed 14 days after cisplatin administration (2 days after appearance of the first purpura lesion). The rheumatoid factor showed no marked changes. The levels of complement components C3 and C4 were below the reference value in the test performed on day 14.

During the hospitalization, the general condition of the patient was good, with a fever only present on days 15 and 16, ranging from 100.22 °F to 100.76 °F, and no presence of arthralgia on any day. The palpable purpura occurred only in the lower limbs, and did not spread to any other site.

Twenty-five days after cisplatin administration and 2 days after discharge from hospital, the chemotherapy regimen was changed to carboplatin AUC 5 (total dose of 375 mg) owing to impaired renal function and the possibility of pharmacodermia (leukocytoclastic vasculitis due to cisplatin administration). After carboplatin administration, the patient had no more episodes of purpura.

## Discussion and conclusions

Leukocytoclastic vasculitis is a type of vasculitis in which the deposition of immune complexes occurs from approximately 7–10 days after exposure to the causative antigen [3]. The patient in the current case reported palpable purpura in the lower limbs 12 days after the high-dose cisplatin infusion, which could indicate leukocytoclastic vasculitis related to drug exposure.

A fresh lesion biopsy, 24–48 h after the first manifestation, usually detects features including fibrinoid necrosis, neutrophil infiltration of the dermal small blood vessel walls, extravasation of erythrocytes, disruption of the vessel wall, endothelial swelling, and nuclear debris, which are typical of leukocytoclastic vasculitis. It is important to highlight that the presence of eosinophils may suggest urticarial or drug-induced vasculitis [6]. A skin biopsy was performed in the current patient and the results were in accordance with the typical characteristics of leukocytoclastic vasculitis.

The first diagnosis hypothesis was meningococcemia, which is a life threatening infection caused by *Neisseria meningitidis*, a gram-negative bacterium. The gold standard for diagnosis of meningococcemia is the isolation of the bacteria from sterile blood fluid such as cerebrospinal fluid and blood [7]. No bacteria were found in the patient’s blood and cerebrospinal fluid, so this hypothesis was discarded.

Another possible diagnosis was Henoch-Schölein Purpura (HSP), also known as IgA vasculitis, usually found in the pediatric population. The immunofluorescence direct test exhibited a negative pattern for IgA, excluding the possibility of HSP. Moreover, some clinical characteristics of the patient did not correspond to those of HSP [8].

Paraneoplastic syndromes are signs and symptoms occurring in different types of cancers that are not directly related to tumor invasion. Lung, urinary tract or gastrointestinal tumors, or hematologic malignancies more frequently cause paraneoplastic leukocytoclastic vasculitis. The main symptoms are palpable purpura on the lower extremities with a burning sensation, pain and pruritus, fever, and malaise [9]. The patient was diagnosed with moderately differentiated head and neck squamous cell carcinoma located in the larynx, which is not the most common type of tumor to cause paraneoplastic syndrome. Of the common symptoms of paraneoplastic syndrome, the patient only had palpable purpura.

In relation to other forms of vasculitis, the patient was tested for rheumatoid factor, C3 and C4 levels, antinuclear antibody, cryoglobulin, ANA and ANCA, and they were all under the laboratory normal range or non-reactive excluding some forms of vasculitis, including ANCA vasculitis, cryoglobulinemic vasculitis, and vasculitis associated with systemic diseases.

Some previous studies have revealed a possible link between leukocytoclastic vasculitis and the administration of omeprazole, acetaminophen, and dexamethasone. One of these studies reports that the administration of omeprazole led to leukocytoclastic vasculitis, confirmed by biopsy, a month after the beginning of the therapy [10]. Frequently used to treat leukocytoclastic vasculitis, dexamethasone can also be responsible for its development. One previous report describes this form of vasculitis in a patient using eardrops with dexamethasone, trimethoprim, and polymyxin B [11].

The current patient had been taking omeprazole, acetaminophen and dexamethasone for over 2 months and had not shown any symptoms; therefore, the possibility of an association between the previous use of these drugs and the development of leukocytoclastic vasculitis was excluded.

For the antiemetic therapy, 0.04% of patients reported leukocytoclastic vasculitis after metoclopramide was co-administered with other drugs. In total 0.03% of patients taking ondansetron co-administered with other drugs also reported leukocytoclastic vasculitis [12]. Regarding dimenhydrinate and mannitol, there are no described cases of leukocytoclastic vasculitis [13]. The current patient received ondansetron with carboplatin infusion and metoclopramide following carboplatin infusion. These two medications were administrated again and the patient did not experience any reaction.

Therefore, the chemotherapeutic agent cisplatin is probably the most likely agent to be responsible for the leukocytoclastic vasculitis in the current patient. The time between the first exposure and the development of the symptoms is in accordance with the literature, the lesion regressed when no new exposure to the agent occurred, and there are other cases linking cisplatin and leukocytoclastic vasculitis. According to the website eHealthMe, 0.05% of patients reported leukocytoclastic vasculitis after cisplatin administration. The time between the development of the leukocytoclastic vasculitis and drug administration was less than one month in 75% of the cases. The medical analysis website also characterizes the most common patient to develop the condition as being male, >60 years old, suffering from non-small cell lung cancer, receiving dexamethasone and carboplatin, and experiencing fever. It was also reported that other types of diseases related to the development of leukocytoclastic vasculitis were squamous cell carcinoma, rash follicular, mesothelioma, and lung adenocarcinoma, whilst larynx carcinoma is not specifically mentioned [12].

The Naranjo Algorithm is a scale used to standardize causality assessment for adverse drug reactions [14]. Applying the Naranjo algorithm to this case, we obtained a score of 4 that classifies our adverse drug reaction as possible, since the reaction followed a reasonable temporal sequence after the drug administration, the reaction was confirmed after the drug withdrawal and could not be explained by the patient’s clinical state.

In conclusion, we believe that the leukocytoclastic vasculitis that developed in the current patient is due to exposure to cisplatin. Our patient had palpable purpura in the lower limbs 12 days after the cisplatin administration, a biopsy was performed confirming the diagnosis, the lesion regressed, and the patient did not have a second exposure to the chemotherapeutic agent due to this reaction and decrease of renal function. To the best of our knowledge, this is the first case report associating high-dose cisplatin therapy with leukocytoclastic vasculitis, and clinicians should be aware of this potential effect for better case management and diagnosis.

## Abbreviations

ANA: Antinuclear antibodies
ANCA: Anti-neutrophil cytoplasmic antibody
BUN: Blood urea nitrogen
CRP: c-reactive protein
CSF: Cerebrospinal fluid
ESR: Erythrocyte sedimentation rate
HSP: Henoch-Schölein Purpura
